# Time-course transcriptome analysis of host cell response to poxvirus infection using a dual long-read sequencing approach

**DOI:** 10.1186/s13104-021-05657-x

**Published:** 2021-06-24

**Authors:** Zoltán Maróti, Dóra Tombácz, István Prazsák, Norbert Moldován, Zsolt Csabai, Gábor Torma, Zsolt Balázs, Tibor Kalmár, Béla Dénes, Michael Snyder, Zsolt Boldogkői

**Affiliations:** 1 Department of Pediatrics, Faculty of Medicine, University of Szeged, Szeged, Hungary; 2 Department of Medical Biology, Faculty of Medicine, University of Szeged, Szeged, Hungary; 3 Department of Genetics, School of Medicine, Stanford University, Stanford, CA USA; 4 Veterinary Diagnostic Directorate of the National Food Chain Safety Office, Budapest, Hungary

## Abstract

**Objective:**

In this study, we applied two long-read sequencing (LRS) approaches, including single-molecule real-time and nanopore-based sequencing methods to investigate the time-lapse transcriptome patterns of host gene expression as a response to Vaccinia virus infection. Transcriptomes determined using short-read sequencing approaches are incomplete because these platforms are inefficient or fail to distinguish between polycistronic RNAs, transcript isoforms, transcriptional start sites, as well as transcriptional readthroughs and overlaps. Long-read sequencing is able to read full-length nucleic acids and can therefore be used to assemble complete transcriptome atlases.

**Results:**

In this work, we identified a number of novel transcripts and transcript isoforms of *Chlorocebus sabaeus.* Additionally, analysis of the most abundant 768 host transcripts revealed a significant overrepresentation of the class of genes in the “regulation of signaling receptor activity” Gene Ontology annotation as a result of viral infection.

**Supplementary Information:**

The online version contains supplementary material available at 10.1186/s13104-021-05657-x.

## Introduction

Vaccinia virus (VACV) the prototypic member of poxviruses, contains a large, double-stranded DNA molecule encoding more than 200 protein-coding genes.

Long-read sequencing (LRS) has now become a mainstream approach in transcriptome studies. LRS is superior over short-read sequencing (SRS) in several respects, including their capability to detect full-length transcripts, which allows the efficient identification of long multicistronic RNA molecules, alternatively transcribed and processed transcripts, and transcriptional overlaps. Currently, two LRS approaches are available from Pacific Biosciences (PacBio) and from Oxford Nanopore Technologies (ONT). Our research group applied both approaches for the study of the transcriptomes of various viruses, including herpesviruses [1–7], baculoviruses, [8], circoviruses [9], retroviruses [10], asfariviruses [11], and poxviruses [12]. These investigations identified a large number of novel transcripts, transcripts isoforms, polycistronic RNA molecules, and transcriptional overlaps.

The effect of viral infection on the host cell transcriptome has been studied in various organisms using SRS [13] and LRS [14]. In this study, we report the time-course analysis of the effect of VACV infection on the transcriptome of African green monkey cells. This work demonstrates the general utility of LRS for the quantitative temporal analysis of gene expression.

## Main text

### Materials and methods

#### Cells, viruses, infection

African green monkey (*Chlorocebus sabaeus*) kidney fibroblast cells (CV-1; from ATCC, USA) were used for the propagation of the Western Reserve strain of VACV. Cells were incubated at 37 °C for 1, 2, 3, 4, 6, 8, and 12 h in a humidified atmosphere containing 5% CO_2_. Following incubation, media were removed and the cells were rinsed with serum-free RPMI 1640 medium and were subjected to three cycles of freeze–thawing.

#### RNA purification

Total RNA was extracted from the viral-infected cells using Macherey–Nagel RNA kit (Düren, Germany) according to the manufacturer’s recommendations. Polyadenylated fractions were isolated from total RNAs using Oligotex mRNA Mini Kit (Qiagen, Hilden, Germany) following the Spin-Column Protocol [1–12].

#### Library preparation

The libraries for sequencing were generated from polyA(+) RNA fractions. For PacBio libraries, the SMARTer PCR cDNA Synthesis Kit (Clontech, Mountain View, California, United States) and the “PacBio Isoform Sequencing (Iso-Seq) using Clontech SMARTer PCR cDNA Synthesis Kit and No Size Selection protocol” were used. The ONT 1D strand-switching cDNA ligation protocol (SSE_9011_v108_revS_18Oct2016, Pacific Biosciences, Menlo Park, California, United States) was used to produce libraries for nanopore sequencing. The details were described in our earlier publications [12, 15, 16].

#### Data processing

Read of inserts (ROIs) from the Sequel raw reads were generated using SMRT Link5.0.1.9585. MinION base calling was performed using the Albacore software v.2.0.1 (Oxford Nanopore Technologies, Oxford, UK). The ONT’s Guppy v.3.6.0 (Oxford Nanopore Technologies, Oxford, UK) was also used for basecalling with the aim to validate the data.

#### Transcriptome profiling of CV-1 cells

The *C. sabeus* reference genome (GCF_000409795.2) was used for aligning of MinION reads. We excluded MAPQ = 0 and secondary and supplementary alignments from downstream analyses. Primary alignments mapping to the VACV genome were counted separately. Reads that were aligned to the host genome were associated with host genes according to the *C. sabeus*_1.1_top_level.gff3 genome coordinates. Only reads that matched the exon structure of reference genes (using a ± 5-bp window for matching exon start and end positions) were counted. Gene counts were normalized to total valid read counts that were mapped to the host genome to identify abundant housekeeping genes that were not influenced by virus–host interactions during the experiment. Geometric means were calculated for the top 16 housekeeping genes (Table 1). A coefficient of variation of < 0.2 was used to normalize gene counts. Our criteria for statistical analyses of gene expression included > 60 normalized total gene counts for the six-time-point data. To classify genes by their expression profiles in the examined time series, we transformed normalized gene counts to a relative scale where the highest expression time point had a value of 1.0 (100%). We performed clustering using the k-means algorithm of the basic statistics package of R (v.3.5) (https://cran.r-project.org/bin/windows/base/old/3.5.0/). The optimal number of clusters was determined using Calinski-Harabasz criteria [17] with the cascade KM algorithm of the vegan (v.2.5-4) R package (Additional file 1: Fig. S1). Clusters were visualized using the heatmap (v.1.0.2) R package. Because few host gene reads failed to enable characterization of host gene expression at the later time points (8–12 h p.i.), host gene expression was only analyzed until 6 h p.i. We clustered the genes into five subcategories according to normalized gene expression profiles. We identified four categories for which gene expression profiles changed during the experiment (Additional file 1: Fig. S2). However, because clustering forces genes into categories, we identified the subset of genes with the most typical expression profiles for each clusters. Thus, in each cluster we plotted mean expression levels for different sampling times using ggplot2 (v.3.1.0, stat_smooth algorithm using LOESS method).

**Table 1:** List of the highly expressed housekeeping genes

GENE	IogFC	logCPM	PValue
RPS18	− 0.3518	12.5098	0.6159
UBL5	− 0.7340	11.0887	0.3131
RPL27	0.0033	12.7196	1.0000
RPS7	− 0.2850	11.4576	0.6914
EEF1A1	− 0.4382	14.8806	0.5136
TPM1	0.0224	11.6027	0.9875
CST3	− 0.7687	13.7938	0.2562
RPL5	− 0.3580	10.6877	0.6357
MYL12B	− 0.2333	10.3555	0.7995
ATP5A1	− 0.7120	11.2808	0.3149
RPL23	− 0.2121	11.1666	0.7709
LOC103217585	0.2172	10.5229	0.7753
RPS5	− 0.2897	10.4022	0.7166
RPL27A	− 0.2296	10.6476	0.7781
RPL17	− 0.6785	11.8542	0.3315
RPS14	− 0.5895	12.3067	0.3968

The scores for each gene were calculated on the basis of expression profiles to represent the alteration of expression level between sampling points that showed the greatest difference in every cluster. Based on this scores, we identified the most characteristic genes in all clusters falling within the range between the top score and the top score–1 SD (Additional file 5: Table S1). Using the identified subset of genes, we performed overrepresentation analyses for the most characteristic genes in each cluster using 768 highly expressed genes as references with the PANTHER (v.14.1 using the 2018_04 dataset release) [18] software tool. We analyzed Gene Ontology biological processes with false discovery rates of < 1.

## Results

In this work, the time-varying transcriptome of CV-1 cells were profiled applying LRS datasets. We used Albacore for basecalling. The Guppy basecaller was also run: we found almost perfect match between the results generated by the two toolkits [15]. The obtained datasets were used to determine the VACV transcripts [15] using the LoRTIA pipeline that was developed by our group [19]. Herein, we used a workflow and a pipeline for transcriptome profiling of LRS datasets that were also developed in our laboratory [4, 6, 20].

MinION sequencing yielded 964,775 host reads (average mapped read length: 583 nts). Sequel sequencing generated 439,330 host ROIs (average mapped read length: 1368 nts). The PacBio MagBead loading protocol selects fragments of less than 1 kb [21]. Dynamic gene expression profiles were classified by transforming normalized gene counts to a relative scale where the highest expression time point had a value of 1.0 (100%).

### ‘Static’ host transcriptome

**Fig. 1:**
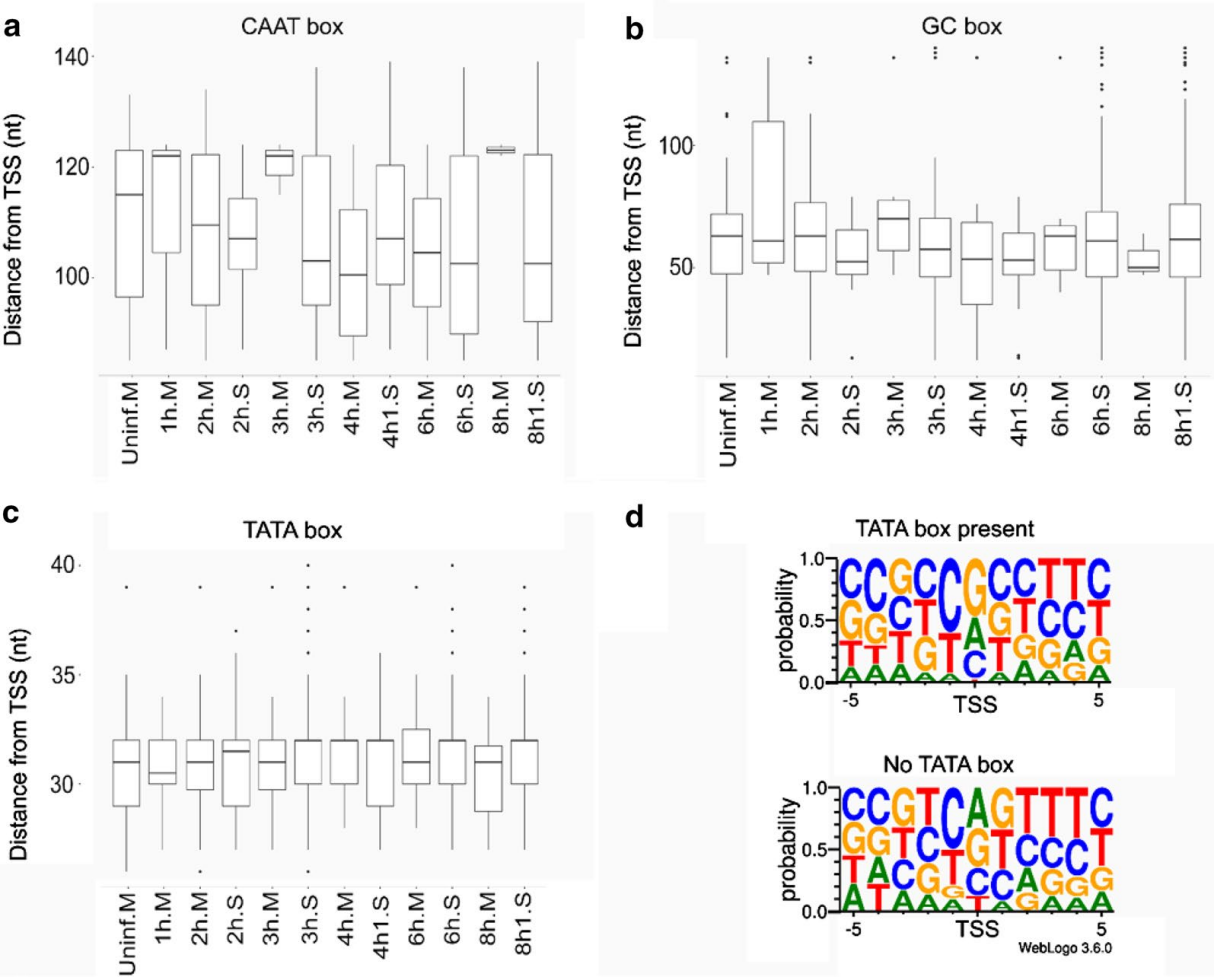
The distance of the host promoter elements from the TSS or the transcription initiator sequence.** a–c** Letter ‘M’ following the sample name indicates MinION sequencing, whereas letter ‘S’ indicates Sequel sequencing. The horizontal lines in the box plots represent the median distance of the given sample. **d** The transcriptional initiator region carries a BBCABW (B = C/G/T, W = A/T) initiator consensus sequence when no TATA box is present upstream of the TSS, whereas the consensus is missing in TSSs with a TATA box

Using the LoRTIA toolkit, we annotated a total of 478 transcription start sites (TSSs), 2011 transcription end sites (TESs), and 24,574 splice junctions, each represented by at least 10 reads (Additional files 6, 7: Tables S2, S3). Analyses of sequence regions upstream of TSSs revealed 43 canonical CAATT boxes (mean distance: 104.913 nt; standard deviation (sd): 15.306), 880 canonical GC boxes (mean distance: 60.095 nt; sd: 33.374), and 80 canonical TATA boxes (mean distance: 31.13 nt; sd: 2.966). It was demonstrated that initiator elements surrounding TSSs of human genes are more likely to be present if the transcript lacks a TATA box upstream of its TSS [22]. In our analyses, the BBCABW (B = C/G/T, W = A/T) initiator consensus was more pronounced around TSSs of TATA-less genes than around those with TATA boxes upstream of their TSSs (Fig. 1).

Specifically, 1,849 TESs contain canonical upstream poly(A) signals (average: 26.681 nt; sd: 9.340), whereas ± 50-nt regions contain U-rich upstream and G/U-rich downstream elements (Additional file 2: Fig. S2). A total of 12,287 introns were annotated: 12,215 of these contained canonical GT/AG, 65 had GC/AG, 7 had AT/AC splice junctions. Those LoRTIA transcripts were accepted that were identified from at least two techniques and in three samples. The average transcript length was 693.661 nt (sd: 962.62), no significant deviations were observed during infection. Lengths of 5’-UTRs deviated around a mean of 52.956 nt (sd: 75.903), whereas the 3’-UTRs had a mean length of 295.219 (sd: 431.480) (Additional file 3: Fig. S3). Transcripts represented by less than ten reads were excluded. These assessments revealed a total of 758 transcript isoforms, 207 with TSSs and TESs in ± 10-nt intervals of previously annotated transcripts, 692 length isoforms, and 66 alternatively spliced isoforms. In total, 239 mRNA length isoforms differed from previously annotated transcripts in their TSS positions [including those with TSSs downstream of respective translation initiation sites (TISs)], 19 in their TES positions, and 56 in both. Altogether 31 transcript isoforms were found with TSSs downstream of previously annotated TISs, and contained curtailed forms of open reading frames (ORFs). These RNAs might code for N-terminally truncated forms of canonical proteins. A total of 177 transcripts were annotated as non-coding. The present non-coding RNAs include either isoforms of previously annotated ribosomal RNAs, or are truncated mRNAs lacking ORFs (Additional file 8: Table S4.)

### Temporal response of the host transcription to VACV infection

**Fig. 2:**
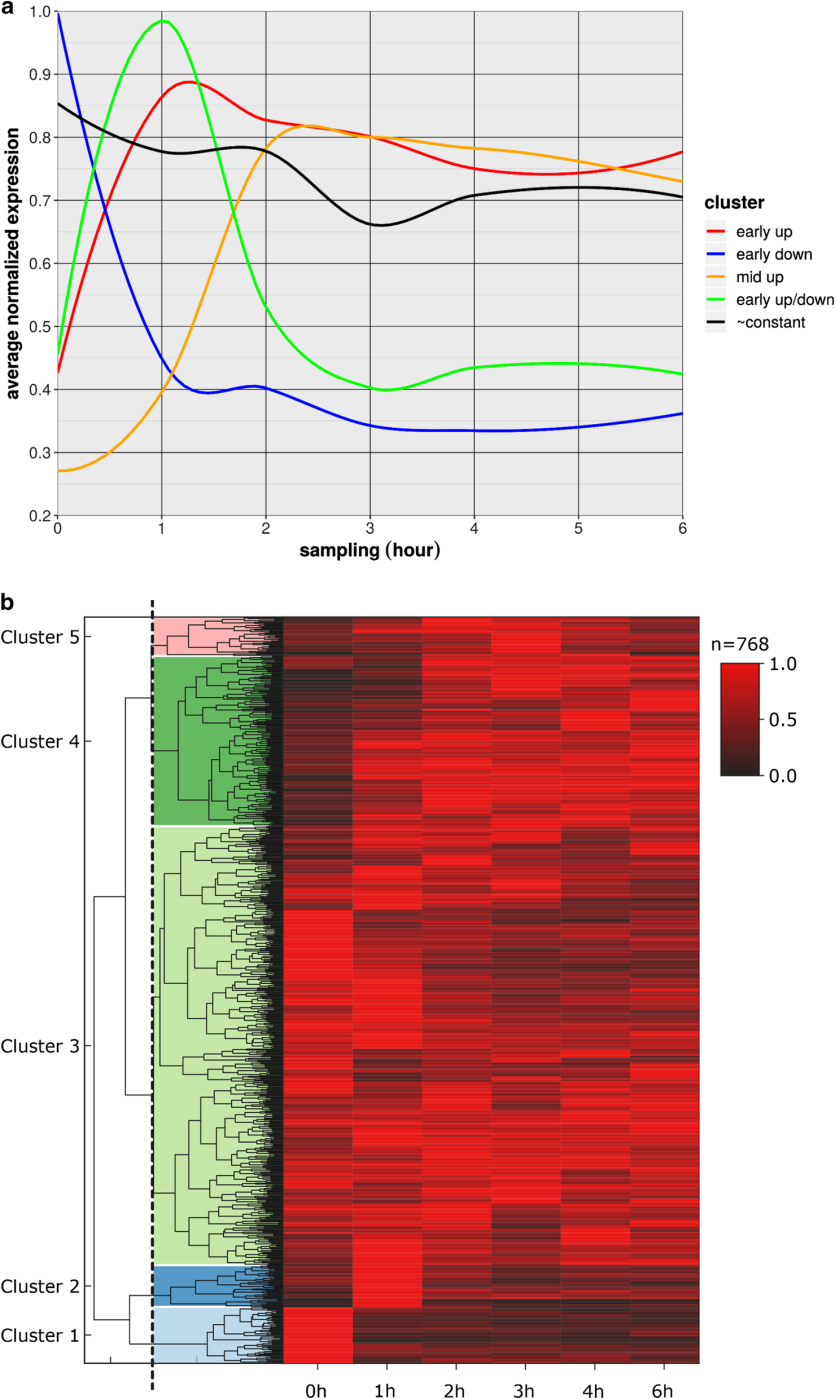
Expression changes of the highly abundant host genes during viral infection. **a** The expression pattern of the host gene clusters. **b** Heatmap representation of the five distinct host gene clusters

The analysis of VACV infection on host transcription revealed relatively few differentially expressed genes [23, 24]. A recent proteomic study also showed that VACV infection affects very few host genes [25]. We categorized five distinct clusters of 768 highly expressed host genes with respect to their responses to viral infection. Among *early up genes*, no or very low expression levels were observed before virus infection, but consistently high expression was observed at all later sampling points*. Early down transcripts* were highly expressed before virus expression and were then constantly absent or had low expression levels at later time points. *Early up/down transcripts* had no or low expression before virus infection, high expression from 1 h p.i. and no or low expression at later sampling points. *Mid up transcripts* had no expression before virus infection and peaked and plateaued at 2 or 3 h p.i. *Constant transcripts* had no significant changes in relative expression levels over the course of our experiments (Fig. 2). We assessed expression patterns of the best characterized gene clusters using GO (Additional file 9: Table S5), and found significant overrepresentation of the GO process “regulation of signaling receptor activity” by genes that were highly expressed in the early stages but were not or only slightly expressed in the late infection phases. Most of the present clusters were not significantly enriched in genes of specific biological processes, although many genes that were upregulated during viral infection were found to play roles in cell division or in “positive regulation of viral life cycle”. Moreover, some genes that were downregulated upon viral infection were annotated to “cell growth” and “mesenchymal differentiation” categories.

## Discussion

We employed LRS platforms for the kinetic analysis of African green monkey transcripts upon VACV infection. Sequencing data were annotated using the LoRTIA pipeline. To avoid annotation of non-specific reads as transcripts, we applied additional filtering of the obtained data using stringent criteria [15], but a certain fraction of excluded reads may represent true transcripts. Our analyses revealed 758 host transcript isoforms. Although partially degraded RNAs are captured by LRS [26], we observed relatively constant mean mapped read lengths from host RNA before and during viral infection. We identified a subset of genes with distinct gene expression profiles during viral infection. Although, only a single class of genes showed significant over representation in the „regulation of signaling receptor activity” GO biological process, we note that this analysis was limited to the study of the most abundantly expressed 768 genes.

In sum, this work identified a large number of host RNAs and transcript isoforms, and revealed a significant overrepresentation of genes in the “regulation of signaling receptor activity” GO annotation in virally infected cells. This study also demonstrates the value of LRS in time-course characterization of the transcriptomes in any organism.

### Limitations

While this work could not cover the full gene sets of the host’s molecular pathways, we think that the expression changes of the identified genes nevertheless are likely the result of virus infection or related to the host’s response to infection. The limitation of this study is the relative low data coverage obtained by both sequencing methods.

## Supplementary Information

**Additional file 1: Figure S1**. Optimal number of clusters based on Calisnki-Harabasz (CH) criterion. The plot on the left shows how each of the genes are partitioned with an increasing number of clusters. On the right, the maximum CH index (for 5 clusters) is shown.

**Additional file 2**: **Figure S2. **The vicinity of the host TESs. (a) Nucleotide distribution surrounding the TESs of *Chlorocebus aethiops* were visualized using WebLogo showing canonical sequences signaling RNA cleavage and polyadenylation. (b) The distance of the host’s polyadenylation signals from the TESs in the uninfected and the p.i. sample. The letter ‘M’ following the sample name indicates MinION sequencing, while letter ‘S’ Sequel sequencing. The horizontal lines in the box plots represent the median distance for the given sample. No significant change in the distance of TESs were observed during the viral infection. The TES positions were determined using the LoRTIA toolkit.

**Additional file 3: Figure S3**. Transcript and UTR lengths of the host (*C. aethiops*) (a) Length of transcripts in the uninfected and each p.i. samples. (b, c) Length of the 5’ and 3’ UTRs were calculated using ti.py in uninfected and each p.i. samples. Letter ‘M’ following the sample name indicates MinION sequencing, and the letter ‘S’ indicates Sequel sequencing. Horizontal lines in the box plots represent median transcript length of the given samples. Transcripts were annotated using the LoRTIA software suit.

**Additional file 4: Fig. S4**. The transcript isoform categories used in this study and their abbreviations.

**Additional file 5: Table S1. **Clustering of the 768 most active host genes based on their expression during VACV infection. Relative expression values at each p.i. time point are normalized to the highest value for a given gene. Genes that were most characteristic of each cluster are highlighted in gray.

**Additional file 6: Table S2. **Transcriptional start and end sites of the host (*C. aethiops*) RNAs. Read counts, distances between GC-, CAAT- and TATA-boxes and TSSs, the sequence of these features, the distance between polyadenylation signals and TESs, and sequences of ± 50-nt regions of TESs are shown. The letter ‘M’ following the sample name indicates MinION sequencing and the letter ‘S’ refers to Sequel sequencing. The TSS and TES sequences were identified using the LoRTIA software suit.

**Additional file 7: Table S3. **Introns of the host transcripts. Splice donor and acceptor positions are shown with read counts and the sequences of the splice junctions. Letter ‘M’ following the sample name indicates MinION sequencing, while letter ‘S’ refers to Sequel sequencing. The introns were determined using the LoRTIA pipeline.

**Additional file 8: Table S4. **Transcript isoforms of host cells. Read counts, category abbreviations, length of transcripts, and length of 5’ and 3’ UTRs are shown. Abbreviation of categories is defined in Supplementary Fig. 4. Transcript isoforms were determined using the LoRTIA toolkit and were categorized using the ti.py script.

**Additional file 9: Table S5. **Overrepresentation analysis of host gene expression levels. The first column contains clusters of host genes and numbers of genes that were characteristic of the cluster (in parentheses). GO biological processes (bold) that had the lowest false discovery rates values are presented with numbers of genes in clusters and numbers of genes in the reference dataset (in parentheses). The genes belonging to GO processes are also listed.

## Data Availability

Raw datasets are available in European Nucleotide Archive: PRJEB26430.The LoRTIA pipeline is available at GitHub: https://github.com/zsolt-balazs/LoRTIA.

## Abbreviations

LRS: Long-read sequencing
VACV: Vaccinia virus
SRS: Short-read sequencing
PacBio: Pacific biosciences
ONT: Oxford Nanopore Technologies
CV-1: African green monkey fibroblast cells
*C. sabaeus*: *Chlorocebus sabaeus*
*C. aethiops*: *Chlorocebus aethiops*
TSS: Transcription start site
TES: Transcription end site
TIS: Translation initiation site
ORF: Open reading frame
