# Supplementary figures and images for: Time-course transcriptome analysis of host cell response to poxvirus infection using a dual long-read sequencing approach
Source: BMC Res Notes. 2021 Jun 24;14:239. doi: 10.1186/s13104-021-05657-x (PMC8223271; doi:10.1186/s13104-021-05657-x)

# K-means partitions comparison

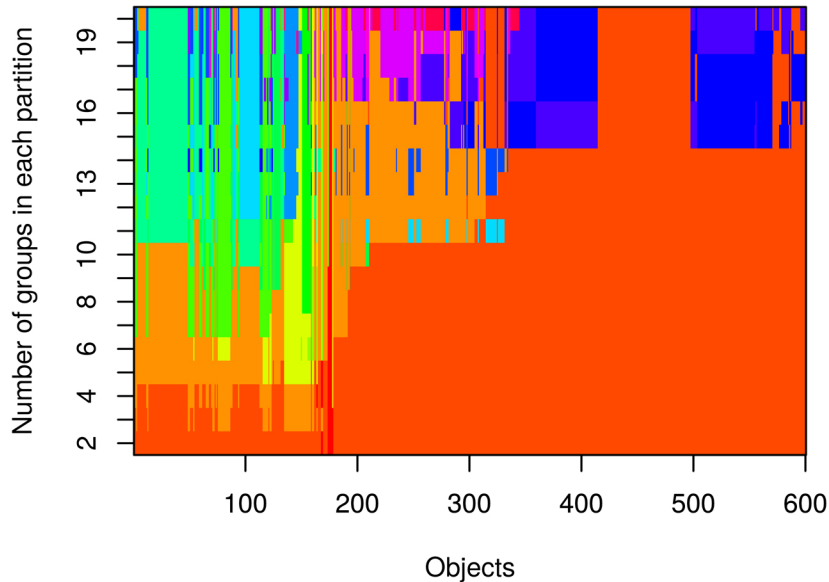

# calinski criterion

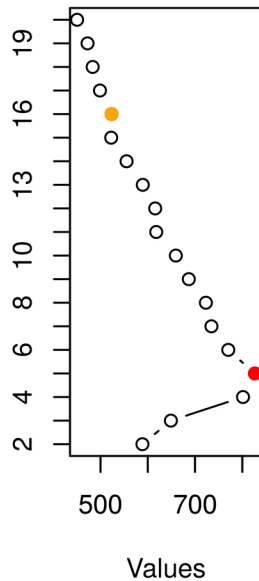

Supplement: Supplementary file 1 — Additional file 1: Figure S1. Optimal number of clusters based on Calisnki-Harabasz (CH) criterion. The plot on the left shows how each of the genes are partitioned with an increasing number of clusters. On the right, the maximum CH index (for 5 clusters) is shown. [file 13104_2021_5657_MOESM1_ESM.pdf]

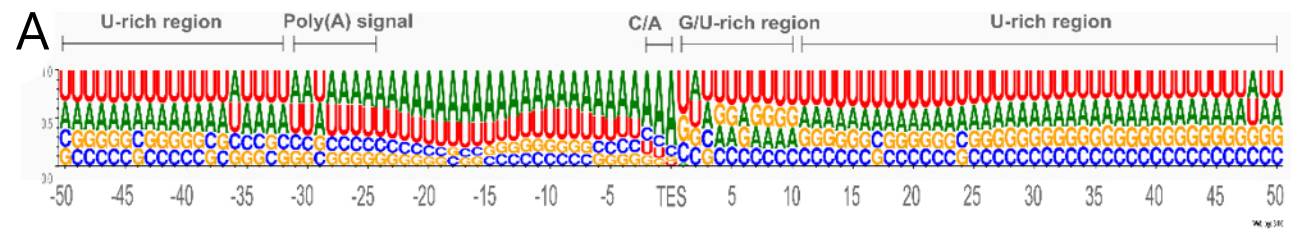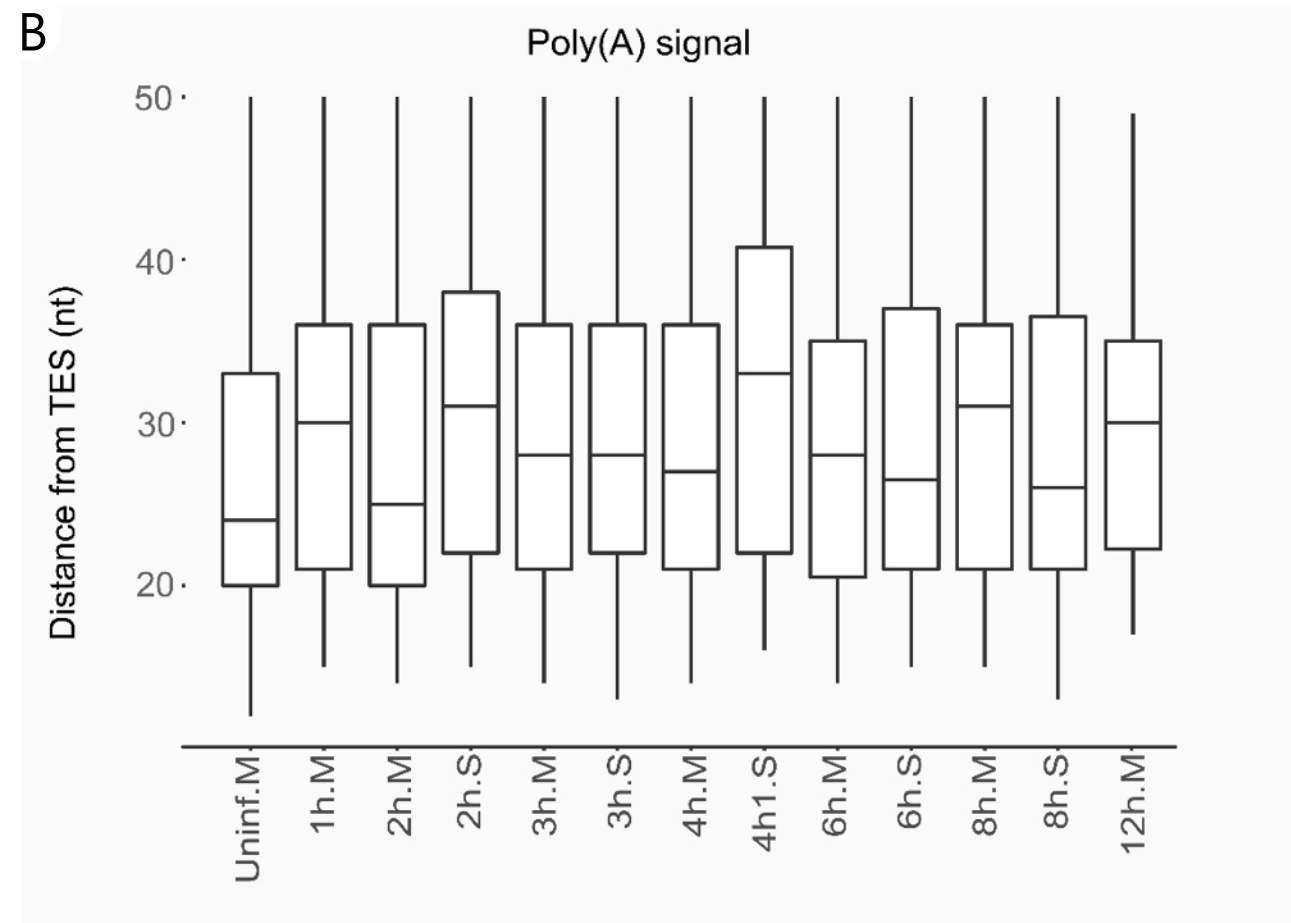

Supplement: Supplementary file 2 — Additional file 2: Figure S2. The vicinity of the host TESs. (a) Nucleotide distribution surrounding the TESs of Chlorocebus aethiops were visualized using WebLogo showing canonical sequences signaling RNA cleavage and polyadenylation. (b) The distance of the host’s polyadenylation signals from the TESs in the uninfected and the p.i. sample. The letter ‘M’ following the sample name indicates MinION sequencing, while letter ‘S’ Sequel sequencing. The horizontal lines in the box plots represent the median distance for the given sample. No significant change in the distance of TESs were observed during the viral infection. The TES positions were determined using the LoRTIA toolkit. [file 13104_2021_5657_MOESM2_ESM.pdf]

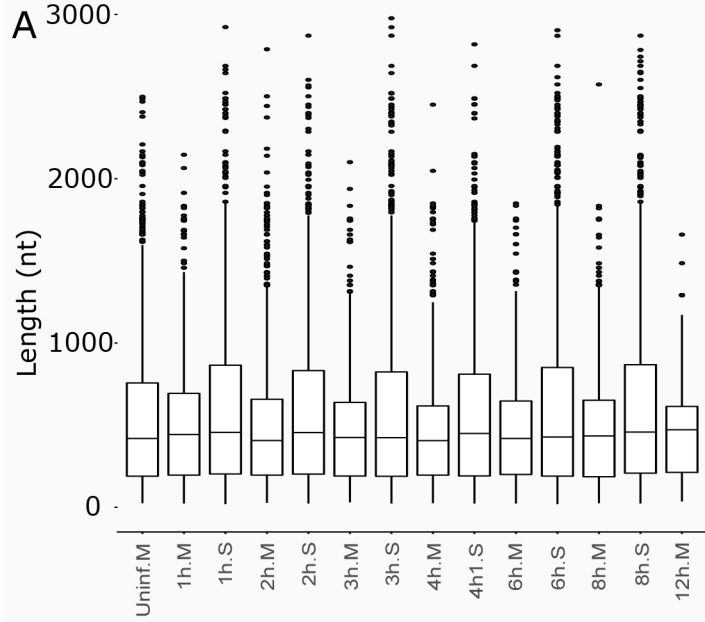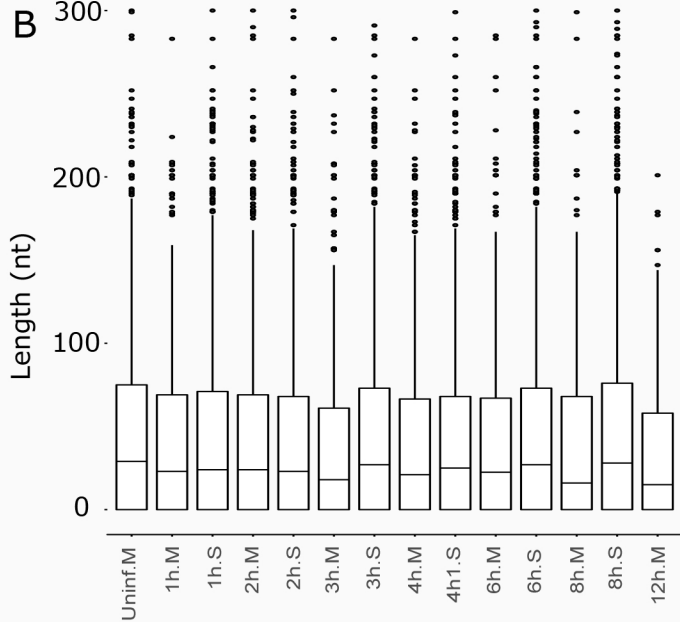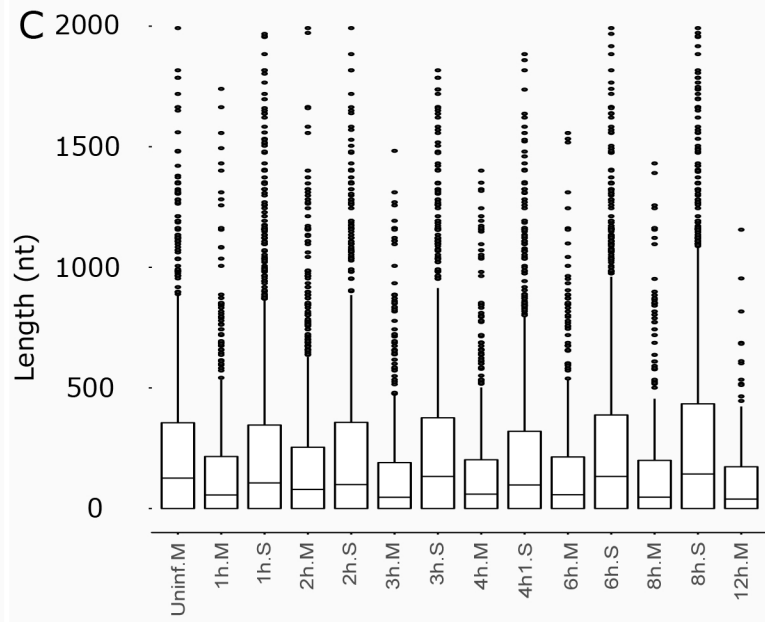

Supplement: Supplementary file 3 — Additional file 3: Figure S3. Transcript and UTR lengths of the host (C. aethiops) (a) Length of transcripts in the uninfected and each p.i. samples. (b, c) Length of the 5’ and 3’ UTRs were calculated using ti.py in uninfected and each p.i. samples. Letter ‘M’ following the sample name indicates MinION sequencing, and the letter ‘S’ indicates Sequel sequencing. Horizontal lines in the box plots represent median transcript length of the given samples. Transcripts were annotated using the LoRTIA software suit. [file 13104_2021_5657_MOESM3_ESM.pdf]

## Types

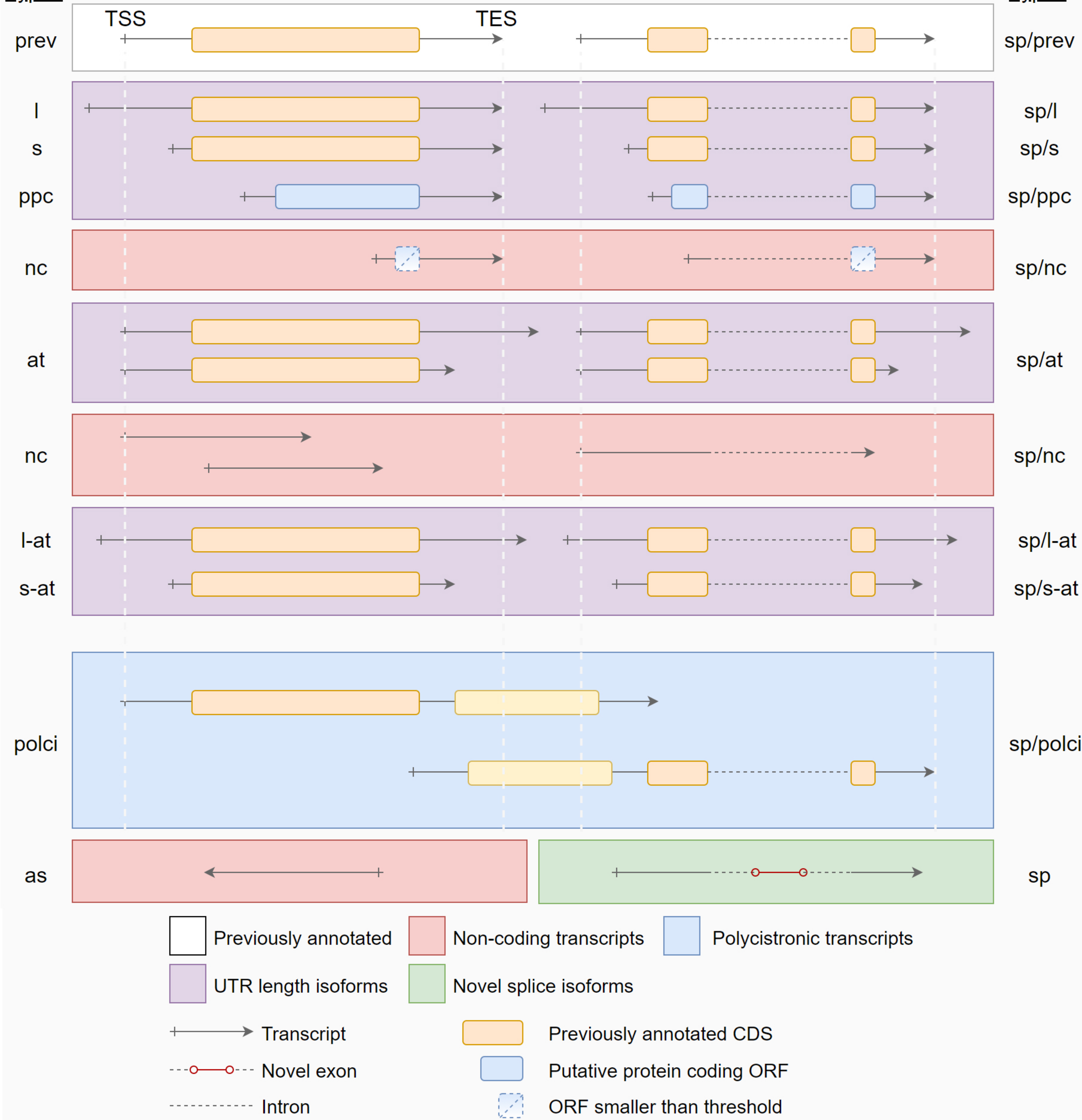

Supplement: Supplementary file 4 — Additional file 4: Fig. S4. The transcript isoform categories used in this study and their abbreviations. [file 13104_2021_5657_MOESM4_ESM.pdf]
